# Morphological atypia and molecular profile of *Plasmodium vivax*: Findings from an outbreak in the Brazilian Amazon[Fn FN1]

**DOI:** 10.1051/parasite/2023039

**Published:** 2023-09-29

**Authors:** Amanda Caroline de Jesus Alves, Ana Cecília Feio dos Santos, José Mário Veloso Peres, José Maria de Souza Nascimento, Danielle Regina Lima Barbosa, Juliana Vasconcelos Figueiredo, Giselle Maria Rachid Viana, Marinete Marins Póvoa

**Affiliations:** 1 Graduate Program in Biology of Infectious and Parasitic Agents, Federal University of Pará Rua Augusto Corrêa, 01 Belém Pará Brazil; 2 Laboratory of Malaria Entomology, Parasitology Section, Evandro Chagas Institute Rodovia BR 316, Km 7 Ananindeua Pará Brazil; 3 Laboratory of Basic Research in Malaria, Parasitology Section, Evandro Chagas Institute Rodovia BR 316, Km 7 Ananindeua Pará Brazil; 4 Multiprofessional Residency Program in Animal Reproduction, Federal Rural University of the Amazon Avenida Presidente Tancredo Neves, 2501 Belém Pará Brazil

**Keywords:** *Plasmodium vivax*, Morphological atypia, Genotyping, Outbreak

## Abstract

This study aimed to perform morphological and molecular analyses of parasites isolated from the blood of malaria-infected individuals during an outbreak in the Microregion of Cametá, State of Pará, Brazilian Amazon. A total of 260 positive samples were identified by microscopy as *Plasmodium vivax*; however, in three samples, forms considered unusual for the species were found and defined as morphological atypia of *P. vivax*. Single *P. vivax* infection was confirmed by qPCR in all samples. Among 256 genotyped samples, the VK247 genotype alone was identified in 255 samples, and the VK210 genotype was found in only one. The study showed that this malaria outbreak was caused by the etiological agent *P. vivax*, and for the first time, morphological atypia was described in isolates circulating in Brazil. Likewise, for the first time, the VK247 genotype was detected predominantly in single infections in an area of the State of Pará, which may suggest a greater circulation of the genotype in the region.

## Abstract (fr)

Cette étude visait à effectuer des analyses morphologiques et moléculaires de parasites isolés du sang d’individus infectés par le paludisme lors d’une épidémie dans la microrégion de Cametá, État du Pará, Amazonie brésilienne. Au total, 260 échantillons positifs ont été identifiés par microscopie comme *Plasmodium vivax* mais dans trois échantillons, des formes considérées comme inhabituelles pour l’espèce ont été trouvées et définies comme des atypies morphologiques de *P. vivax*. Une infection simple à *P. vivax* a été confirmée par qPCR dans tous les échantillons. Sur 256 échantillons génotypés, le génotype VK247 seul a été identifié dans 255 échantillons, et le génotype VK210 a été trouvé dans un seul échantillon. L’étude a montré que cette épidémie de paludisme était causée par l’agent étiologique *P. vivax* et, pour la première fois, une atypie morphologique a été décrite dans des isolats circulant au Brésil. De même, pour la première fois, le génotype VK247 a été détecté principalement dans des infections uniques dans une zone de l’État de Pará, ce qui peut suggérer une plus grande circulation du génotype dans la région.

## Introduction

According to the World Health Organization (WHO), malaria, a parasitic disease caused by protozoa of the genus *Plasmodium*, is a public health problem in tropical and subtropical areas of the world. According to the latest global malaria report, in 2021 there were approximately 247 million cases and 619,000 deaths worldwide [27].

In the Americas, Brazil is the country with the second highest number of malaria cases, where local transmission of *P. vivax, P. falciparum* and *P. malariae* occurs, and *P. vivax* is responsible for more than 80% of cases. In 2022, the country recorded 129,606 cases of the disease, almost exclusively restricted to the Legal Amazon region (99% of all reported cases) [3, 27].

Despite morphological similarities among *Plasmodium* species, with sexual cycle occurring in the *Anopheles* mosquito and asexual cycle in the human host, each *Plasmodium* species has morphological and biological characteristics that impact its epidemiology. For example, vivax malaria presents the possibility of relapses through reactivation of latent hepatic forms (hypnozoites) [30] and the early appearance of gametocytes in the blood circulation, leading to a high potential for disease transmission [1]. Furthermore, by investigating the circumsporozoite protein (CSP), *P. vivax* was genotypically classified into three variants, VK210 [2], VK247 [17] and *P. vivax*-like [15]. Such characteristics, together with sociodemographic, political, and environmental factors in the Amazon region, make it difficult to control malaria, leading to the occurrence of epidemic outbreaks and making it essential to carry out studies that investigate the behavior of the species, especially in endemic areas [23]. Thus, the present work aimed to perform morphological and molecular analyses of parasites isolated from the blood of malaria-infected individuals during an outbreak in an endemic area of the Brazilian Amazon.

## Materials and methods

Individuals with symptoms of malaria who received primary care were recruited from the Public Health Service of the municipality of Cametá in the Microregion of Cametá, State of Pará, Brazilian Amazon, during an outbreak that occurred between 2017 and 2018. Blood samples along with clinical and sociodemographic information were obtained during follow-up epidemiologic surveillance support by the Instituto Evandro Chagas (IEC – Ministry of Health, Brazil). From each individual, 5 mL of peripheral blood were collected by venipuncture to perform the parasite identification techniques. Three slides were prepared for morphological identification, one of only thick smear, stained and read by professionals from the local health unit for patient diagnosis, and two containing thick smear and blood distension, each stained by the team at the study site and sent to the Laboratory of Basic Research in Malaria of the IEC for confirmation of the parasite species and morphological analysis. In addition, part of each blood sample was stored in tubes with EDTA and later centrifuged and divided into cryogenic tubes with packed red blood cells and tubes with plasma, which were frozen at −20 °C and sent to the IEC for analysis by molecular biology techniques. As this was an IEC health surveillance action, and due to the anonymity of the samples, submission to the research ethics committee was not necessary.

The slides were prepared and stained as recommended by the Brazilian Ministry of Health [4]. To prepare the thick smear, the blood was placed on the surface of the slide with the aid of a pipette. Then, with the corner and the first 5 mm of the edge of another slide, the drop was spread to form a rectangle of size and thickness of approximately 1.2 cm^2^. For staining, the Walker method was used, in which red blood cells were dehemoglobinized by a methylene blue phosphate solution for 2 s and then the slides were rinsed with buffered water. Subsequently, the slides were placed facing the surface of a concave staining plate, where a 5% diluted Giemsa alcohol solution was applied (50 μL of the stock solution to 1000 μL in buffered water at pH 7.0–7.2). The slides were in contact with this solution for 10 minutes, then washed with buffered water, dried at room temperature, and stored in slide boxes until transported to the IEC for reading. To make the blood distension, the blood was placed on the slide and, with the narrow edge of a beveled slide in contact with the drop of blood forming a 50° angle, the blood was spread with a rapid movement to form a thin layer without reaching the edge of the slide. The slide was fixed with methyl alcohol for 1 min and, after drying, it was stained using the Giemsa method. In this method, the slide was inverted and placed on a concave staining plate, and a 5% diluted alcoholic solution of Giemsa was poured to come into contact with the blood distension. The slide remained in contact with the stain for 20 min, then washed with buffered water, dried at room temperature, and stored in slide boxes until transportation to the IEC.

The slides were read under an optical microscope using a 100× objective (immersion). The thick smear was used to identify the causative species and to determine the parasitemia, and the blood distension was used to assist in morphological identification. Parasitemia was calculated by counting the number of asexual parasites identified in 200 leukocytes observed in the thick smear, based on a count of 6000 leukocytes/μL and with the aid of a manual counter. Sexual forms (gametocytes) were also counted, and their number per microliter was estimated similarly to asexual forms [4].

To confirm the *Plasmodium* species by molecular techniques, the parasite’s DNA was extracted from samples of packed red blood cells obtained from each individual. For this, a commercial kit ReliaprepTM gDNA Tissue Miniprep System (Promega, Madison, WI, USA) was used, according to the manufacturer’s recommendations.

Then, qPCR was performed on all samples according to the protocol of Rougemont *et al*. (2004) [18], which identifies the four main species of *Plasmodium* (*P. falciparum, P. vivax, P. malariae*, and *P. ovale*). The assay was performed on the StepOnePlus™ Real-Time PCR System (Applied Biosystems, Foster City, CA, USA).

Subsequently, samples with a positive result for *P. vivax* were amplified for the repetitive region of the CSP gene by PCR following the protocol described by Cassiano *et al*. (2011) [6]. The reaction was performed in a Veriti^TM^ 96-Well Thermal Cycler (Applied Biosystems). To visualize the amplified samples, the PCR products were subjected to electrophoresis at 100 V for 50 min together with a 100 bp molecular weight marker Trackit (Invitrogen, Waltham, MA, USA) on an agarose gel with a concentration of 1.5%. Subsequently, the gel was visualized under ultraviolet light and photographed in a photo documentation system, Edas 290^®^ (Kodak, Rochester, NY, USA).

After PCR, *P. vivax* CSP variants were characterized by restriction fragment length polymorphism analysis, following the protocol described by Cassiano *et al*. (2011) [6], with adjustments. The resulting products of the digestion reaction were subjected to electrophoresis at 100 V for 90 min together with a 50 bp molecular weight marker Trackit type (Invitrogen) on a 2.5% agarose gel stained with GelRed 10,000× in water (GelRed Nucleic Acid Gel Stain, Biotium^®^, Uniscience LTDA). Subsequently, the gel was visualized under ultraviolet light and photographed in a photo documentation system (Edas 290^®^). Digestion for genotype VK210 results in bands of 135, 106, 100, 54, 43 and 24 bp; for genotype VK247, bands of 673, 100 and 43 bp; and for *P. vivax*-like, bands of 731, 62 and 41 bp. For statistical analysis of the results, a descriptive analysis was performed by simple counting.

## Results

Initially, 569 individuals with suspected malaria were investigated, of which 260 had a positive result in the microscopic analysis and were included in subsequent analyses. The two slides of all patients were read, and parasitemia values ranged from 24 to 37,500 parasites/mm^3^. Microscopy revealed, by thick smear reading, evolutionary forms characteristic of *P. vivax*. However, during the blood distension reading, forms considered unusual for the species were found in three samples (1%), resembling the morphology observed in infections by other *Plasmodium* species that affect humans.

Although these samples presented morphological forms that could suggest infections by other species, microscopically all infections were identified as *P. vivax*, without mixed infections, due to the predominance of morphological forms typical of the species, in addition to molecular confirmation. Thus, the forms observed were defined as morphological atypia of *P. vivax*, corresponding to parasites in different stages of development, including trophozoites, gametocytes and schizonts.

**Figure 1:**
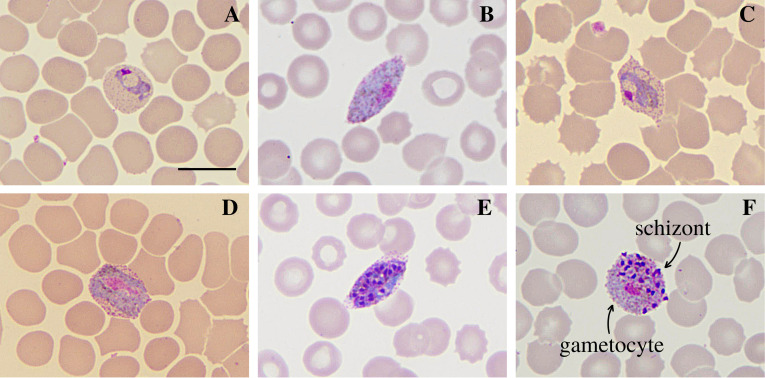
Atypical *P. vivax* morphologies found in sample CAM-185*.* Slides were prepared by the blood distension technique, stained by the Giemsa method, and visualized at 100× magnification under an optical microscope. (A) basket-form trophozoite; (B–C) gametocytes in oval red blood cells; (D) band-form gametocyte; (E) schizont in oval red blood cell; (F) simultaneous infection of schizont (with about 20 merozoites) and gametocyte in a single red blood cell. Bar = 10 μm.

The sample identified as CAM-185 belonged to a 39-year-old individual who had fever in the 3 days preceding the test, had not taken antimalarial medication, and had no history of travel in the previous 30 days. The result found was 8460 V (asexual forms of *P. vivax*), 960 Vg (gametocytes of *P. vivax*)/μL of blood, and qPCR showed Ct = 26.91. Binucleated trophozoites and basket-shaped trophozoites, gametocytes in oval red blood cells and gametocytes occupying the red blood cell in a distended manner were observed, forming a “band” from one end of the cell to the other. Schizonts were also found in oval red blood cells similar to those observed with gametocytes, in addition to the atypical form of simultaneous infection in the same red blood cell by a gametocyte and a schizont with approximately 20 merozoites (Fig. 1).

**Figure 2:**
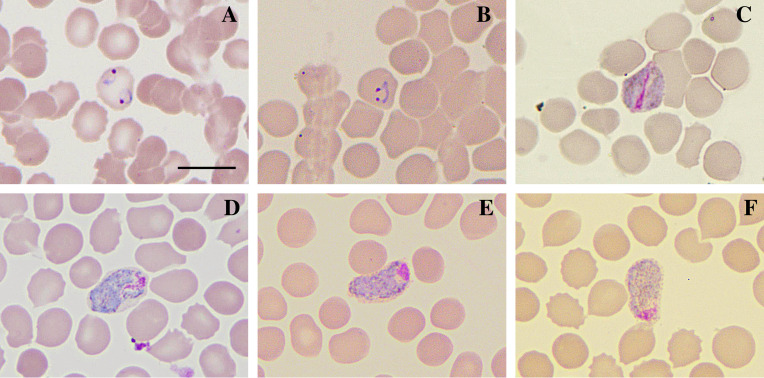
Atypical *P. vivax* morphologies found in the sample CAM-190*.* Slides were prepared by the blood distension technique, stained by the Giemsa method, and visualized at 100× magnification under an optical microscope. (A) multi-infected red blood cell with trophozoites; (B) binucleated trophozoite; (C) band-form gametocyte; (D–F) elongated gametocytes resembling a sausage shape. Bar = 10 μm.

Sample CAM-190 belonged to a 20-year-old individual who had fever for the 4 days preceding the test, had not taken antimalarial medication in the previous 30 days, and had no information regarding travel history, with a parasitemia of 21,450 V, 810 Vg/μL and Ct = 27.14. Erythrocytes with double invasion by trophozoites were found, in addition to trophozoites with double chromatin in several fields of the slide. Elongated gametocytes were also observed, in some cases resembling the crescent shape (sausage), as well as gametocytes distended in red blood cells and gametocytes without a well-defined shape (Fig. 2).

**Figure 3:**
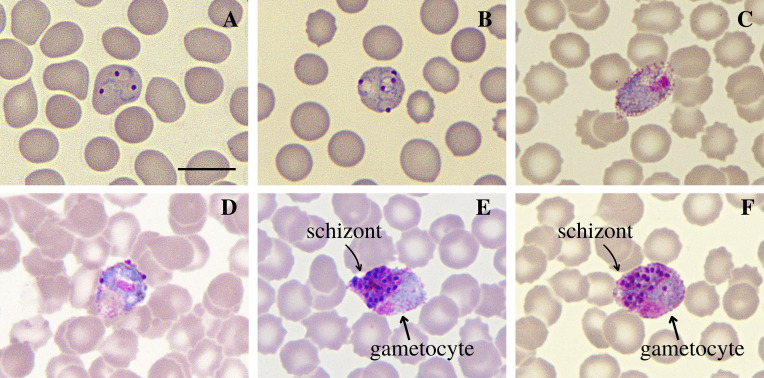
Atypical *P. vivax* morphologies found in the sample CAM-466. Slides were prepared by the blood distension technique, stained by the Giemsa method, and visualized at 100× magnification under an optical microscope. (A–B) multi-infected red blood cells with trophozoites; (C) gametocyte in oval red blood cell; (D) atypical gametocyte with extra nuclei similar to trophozoites nuclei; (E–F) simultaneous infection of schizont (with 19–21 merozoites) and gametocyte in a single red blood cell. Bar = 10 μm.

Sample CAM-466 belonged to a 58-year-old individual who had fever in the 6 days prior to the test, did not use antimalarials in the preceding 30 days, and had a history of travel to a neighboring city that was also in an epidemiological outbreak period. Analysis of CAM-466, whose parasitemia was 11,520 V, 360 Vg/μL and Ct = 25.04, showed red blood cells with multiple invasion by trophozoites, with up to three parasites in the same cell. Gametocytes were also found in oval red blood cells, gametocytes with irregular morphology containing shapes that resemble trophozoite nuclei, in addition to the form of simultaneous infection of a gametocyte and a schizont with approximately 20 merozoites in the same red blood cell (Fig. 3).

**Figure 4:**
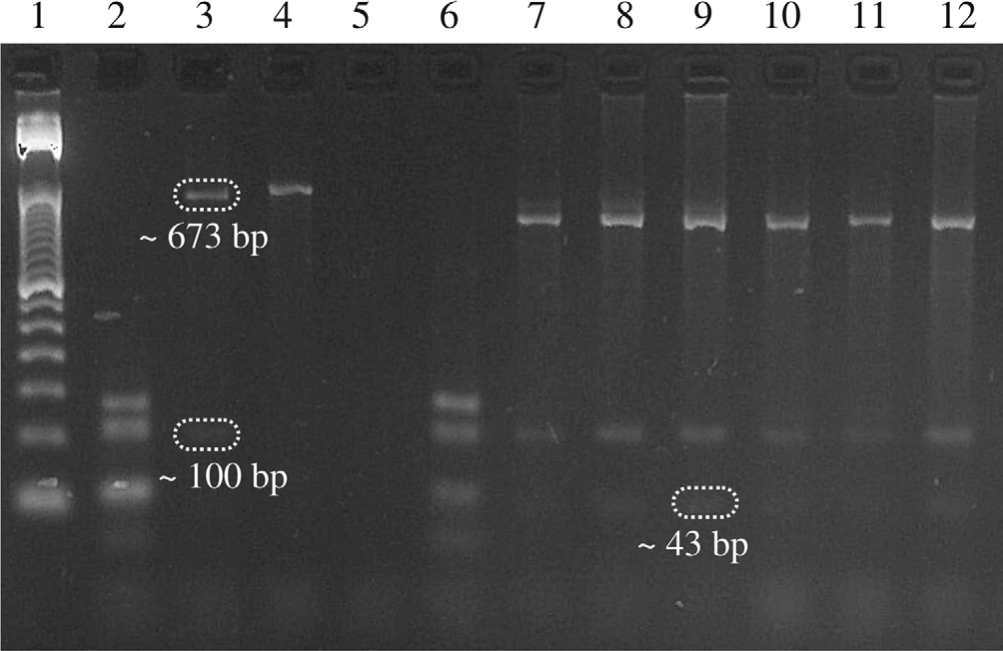
Samples showing a banding pattern compatible with the VK210 and VK247 genotypes after digestion with the AluI enzyme of the amplified product. Electrophoresis in 2.5% agarose gel visualized under ultraviolet light. (1) 50 bp molecular weight marker; (2–4) positive controls of the VK210, VK247 and *P. vivax*-like genotypes, respectively; (5) negative control; (6) single sample positive for the VK210 genotype; (7–12) samples positive for the VK247 genotype, with samples 7, 8 and 9 showing atypical morphology in the microscopic analysis.

The results obtained by microscopy of all 260 samples were confirmed by qPCR, demonstrating that the species responsible for all infections was *P. vivax* alone and that the microscopic diagnosis presented results in agreement with the molecular diagnosis. Of the 260 samples confirmed by qPCR, 256 were amplified and genotyped. Among the 256 samples, in 255, the VK247 genotype alone was identified, including samples that showed morphological atypias. In only one, the VK210 genotype was identified (Fig. 4). The *P. vivax-*like genotype was not found in any of the samples, and likewise, no mixed infections were identified. Thus, there was a predominance of the VK247 genotype in single infections (99.6%).

## Discussion

As microscopic examination of the parasite is considered the gold standard for the diagnosis of malaria and the most used method in Brazil is blood thick smear microscopy [5], it is important to note that in the present study the atypical forms were visualized only in blood distension. Therefore, important morphological details may not be perceived in thick smear, such as atypia that can provide important information about *P. vivax* isolates circulating in Brazil. The importance of using blood distension in association with thick smear in the diagnosis of malaria must be considered, because the inadequate diagnosis of the infecting species can also lead to incorrect treatment of the patients, potentially worsening their clinical condition and serving as an infectious parasite reservoir, further aggravating the epidemiological situation of malaria.

Morphological atypia in infections by *P. vivax* has been reported in some studies and has mainly included multiple infections by trophozoites in the same red blood cell, trophozoites with double chromatin (binucleation), oval-shaped erythrocytes, mature schizonts of small size and a reduced number of merozoites [13]. In the present study, several of these described atypia were found.

Multiple trophozoite infections were found at a high frequency in this study and corresponded to up to three forms in the same red blood cell, which despite the possibility of being found in *P. vivax*, is more commonly observed in *P. falciparum* infections [4, 26]. The observation of multiple infections is the most frequent atypia in *P. vivax* and has been associated with several factors, such as possible mixed infection with *P. falciparum* [12] or simian *Plasmodium* species [25], use of immunosuppressive drugs [21], presence of other parasites [28] or certain strains of *P. vivax* [10, 13], clinical conditions of the patient, and intensity of the parasitemia, as a high degree of parasitemia associated with anemia could facilitate multiple invasions of the same red blood cell [13, 26, 28].

In this study, it is known that all samples were positive for *P. vivax.* Nonetheless, as samples are from a period of epidemic outbreak with a large flow of patients, there is no detailed information on the patients’ clinical statuses, and it is only known that none of them used antimalarials in the 30 days prior to the examination, without information about other medications. Furthermore, the parasitemias found in the three atypical samples were considered moderate, and considering the molecular confirmation performed by qPCR in our study and the epidemiology of simian malaria in Brazil, there is no reason to suppose that these are infections caused by simian parasites, since the atypias described here are not characteristic of any of the species found in the country. Therefore, it was not possible to associate the presence of atypical forms with the factors mentioned above.

In addition to multiple trophozoite infection, the simultaneous infection by a gametocyte and a schizont found here has also been previously reported in a study by Poirriez *et al*. (1991) [13] and another by Mazars *et al*. (1997) [12]. This process of simultaneous development of a sexual (gametocyte) and asexual (schizont) form of a parasite in the same blood cell is known as pseudoparthenogenesis, and images of it are very rarely found [13]; however, it was demonstrated in two patients in our study (Figs. 1 and 3). According to Wang (1970) [26], initially it was believed that a single merozoite was divided into sexual and asexual forms (theory of parthenogenesis). However, it was shown that it was a multiple invasion, giving rise to the so-called parthenogenetic body formed by two parasites in the same erythrocyte with poorly defined membranes separating the gametocyte and the schizont, whose meaning is not well understood.

Another atypia found was the presence of gametocytes occupying the red blood cell in a distended form from one cell end to the other, which was also reported by Mazars *et al*. (1997) [12]. In addition, gametocytes with irregular morphology containing shapes similar to trophozoite nuclei observed in one of the patients (Fig. 3) resembled the multiple infection forms of a gametocyte and 1–3 young trophozoites in the same RBC also found by Tsukamoto (1977) [25]. In both our study and Tsukamoto’s, highly deformed (oval) red blood cells similar to those found in *P. ovale* infections were observed. Considering that the atypia demonstrated by Tsukamoto (1977) [25] occurred both before and after the use of antimalarial drugs and that in the present study, no patient reported the use of these drugs in the period prior to the examination, it is likely that the occurrence of atypia is not related to antimalarial therapy.

The presence of oval red blood cells was also observed by Gautret *et al*. (2001) [8], who reported RBCs containing only trophozoites, whereas we identified gametocytes and schizonts within RBCs. In the study by Gautret *et al*. (2001) [8], microscopic and geographic criteria indicated that it was a case of infection by *P. ovale*. However, the molecular diagnosis revealed the case to be, in fact, an infection by *P. vivax*, leading the authors to consider several factors as causes of atypia. These factors included problems in the preparation of slides that can make the diagnosis difficult, in addition to the possibility that it was a *P. vivax-*like infection which, despite being microscopically identical to *P. vivax*, has a CSP sequence identical to that of *P. simiovale,* a nonhuman primate parasite morphologically similar to *P. ovale*. Concerning this study, all slides were prepared in a standardized way, all samples were from a *P. vivax* epidemic outbreak and molecularly confirmed, and autochthonous transmission of *P. ovale* does not occur in Brazil. Moreover, in the atypical samples, only the VK247 genotype was detected. Therefore, it is unlikely that the factors considered by Gautret *et al*. (2001) [8] triggered the atypia observed here.

Although there is no information about the origin of the atypical forms, to our knowledge, this is the first report of atypia in *P. vivax* isolates in Brazil. However, it is important to consider that atypia was possibly not reported before in Brazil because the main technique used in the country is thick smear microscopy, which does not provide morphological details as effectively as blood distension.

Considering that the atypia found occurred in *P. vivax* isolates circulating in an epidemic outbreak, in addition to the numerous possibilities that can trigger them, it is necessary to carry out studies that can clarify the possible factors that lead to their emergence, and to verify whether there is an influence on the clinical course and treatment of the patient and, consequently, on disease control.

In addition to the atypical forms of *P. vivax*, the VK247 genotype was the most prevalent (99.6%), which differs from previous findings for the Brazilian Amazon, where the VK210 genotype was the most frequent and VK247 was detected only in mixed infections [9, 11, 14, 16, 22]. In a study covering five states of the Brazilian Amazon, Storti-Melo *et al*. (2009) [24] detected the VK247 genotype for the first time in single infections in the country, exclusively in the State of Pará. Santos *et al*. (2016) [19] identified single infections by VK247 in humans and mosquitoes circulating in the municipality of Goianésia do Pará, also in Pará State, which may indicate an adaptation of this genotype in the region and, thus, a change in the dynamics of *P. vivax* genotype distribution. In these two studies, although the VK247 genotype was detected in single infections, the VK210 genotype remained the most frequent, unlike our findings of the highest prevalence of the VK247 genotype. A similar result was found in the State of Roraima, where the VK247 genotype was more frequent than the VK210 genotype. In this same study, a gradual increase in the VK247 genotype was demonstrated in the states of Amapá and Pará [20].

Although the samples in the present study originate from an epidemic outbreak period, the identification of the VK247 genotype in single infections in the Brazilian Amazon is an unusual finding. It is more likely that the outbreaks in the region are caused by the VK210 variant or, at the very least, by mixed genotype infections. This finding may be an indication that the VK247 genotype is showing increased circulation in the region, as suggested by previous studies [19, 20].

Changes in the dynamics of *P. vivax* genotype distribution may be explained by different factors, such as selection by host immune pressure in relation to a particular genotype and/or changes in the presence or prevalence of different species of mosquito vectors susceptible to infection for specific genotypes, but these factors are still not well understood [7, 29].

Considering the prevalence of an unusual genotype in single infections in the Brazilian Amazon region and the reported atypical forms of *P. vivax*, further studies are necessary to clarify the factors that may have caused these events. Additionally, understanding any potential relationships and the possible consequences of these findings is warranted.
